# Supplementary material for: Isolation of an Insulin-Like Receptor Involved in the Testicular Development of the Mud Crab Scylla paramamosain
Source: Int J Mol Sci. 2023 Sep 4;24(17):13639. doi: 10.3390/ijms241713639 (PMC10487528; doi:10.3390/ijms241713639)
Supplement: Supplementary file 1 [file ijms-24-13639-s001.zip › ijms-2579348-supplementary.pdf]

1 ACATGGGAACGGGATGAGCCCAGCGCATAACCTGGCGTCGTAACCCGGCGCCAGGGGTGT

61 TGCGTTTCGGAGGGTCCGGCACGCAGTCTCTCACCACCACCAGAAAAATTACAGTTACCA

121 CCACCACCACTACCACCGCGACATCCACCATCGTCCTCACCACCATCACCACCACCACCA

181 CCACCGCCGTCCACCATCACCAGTACTCAAAGACCCCTGCAGCTTTCACCACCACACGCC

241 CCCTTGGGAAGGGCCACTACGGGCCCCACGCCGCCCTCCTACGCGCCCCTCCGCTGCGGC

301 AGGGGC**ATG**TGGGAGTGGCGGGCATGGGTGCTGGTGGTGCTGGCAGTGCTGGGGGCGGCC

M W E W R A W V L V V L A V L G A A

361 AGGGAGTGCCACGCCCAAGCCCACTACGGGGAAACGTCGACTTCCGCCTCCCTTTGTCCG

R E C H A Q A H Y G E T S T S A S L C P

421 CCAAAAAGAGGCTCCTCTCAGAAAATCTGCAGCAGCATGGATTTGAGGAACAACGTGGAG

P K R G S S Q K I C S S M D L R N N V E

481 GAGCTGGCGAGGCTGTGTCACTGCTCCATCATTGAGGGTTACCTGCATTTCGTCCTCCAG

E L A R L C H C S I I E G Y L H F V L Q

541 GAACAGAACGACAGCGCCTCTGCCGCCAAGCTGAGGGAGTTCACATTTCCTGAACTGCGG

E Q N D S A S A A K L R E F T F P E L R

601 GAAATCACGGAGCACCTTCTCGTGTACAGAGTGTTCAGCCTTACCACCCTCAGGACGCTC

E I T E H L L V Y R V F S L T T L R T L

661 TTCCCCAACCTCACCGTCATCAGAGGGGAGGTGCTCTTCTACAACTACGCCTTAGTGATC

F P N L T V I R G E V L F Y N Y A L V I

721 TACGACACACCTAATCTGGTGGAGGTGGGCCTGGCTTCCCTCACCTCCATCCTCAGAGGA

Y D T P N L V E V G L A S L T S I L R G

781 TCGGTGCGAATAGAGAGGAACCGGCAGCTGTGTCACGTGACCACGGTGGACTGGACTCGC

S V R I E R N R Q L C H V T T V D W T R

841 ATCACCTCCAGGCCACTCACGAAAAACTACATCAACCGGAACAAGCCTGAGTCGGAGTGC

I T S R P L T K N Y I N R N K P E S E C

901 CGCCCCTGCCCTGCTGACATGGGCTGCCCCGCCGCCACACGCTGCGGGCCCGGGCGCTGC

R P C P A D M G C P A A T R C G P G R C

961 TGGGGACCCGGGGCGTGCCAGATTCTGTGTAGGAGCGGGTGTCCGGGTGGATGTGTGGGC

W G P G A C Q I L C R S G C P G G C V G

1021 GGGGAGTGTTGCCACGAAGAGTGTGTCGGAGGCTGCCTCACGCCGGGGAGCCCCAGGACC

G E C C H E E C V G G C L T P G S P R T

1081 TGCCTCGCCTGCAGGAACTTCTGGGACGTGGAGGAGGGCCGCTGCTCCCCCGACTGCCCG

C L A C R N F W D V E E G R C S P D C P

1141 CCTGGGAAACTCAAGGTATCGAGGCACCGGTGCGAGCGGGTGCAGGCGTGTCCCCATGGT

P G K L K V S R H R C E R V Q A C P H G

1201 CGCGCCCTGAGGCTGGACACGCGGGAGTGTGTGTGGGAGTGTCCCGCGGGCCATGAAAAC

R A L R L D T R E C V W E C P A G H E N

1261 ACCACCTCGGCGATCTTTGGGAGGCTGTGTCGCCCCTGCCAAGGTCACCAATGTCCCAGG

T T S A I F G R L C R P C Q G H Q C P R

1321 AGGTGCCCAGGAACTAACGTGAGTAGTGTGAGTCACGCCCAAGCACTGCGAGGCTGCACG

R C P G T N V S S V S H A Q A L R G C T

1381 TTGATCGAAGGAGGCCTCACCATTCTGATCAACGGTGGAGAGAATGTGCAGAAGGAGCTG

L I E G G L T I L I N G G E N V Q K E L

1441 GAGGAGAACCTGAGCACCATCAAGGAGGTGGAGCACTTCGTCAAGGTGTTCCGATCCAAC

E E N L S T I K E V E H F V K V F R S N

1501 CTCACCTCGCTGACCTTCTTCTCCTCCCTCACTCGCGTCGGTGGCCTGGAACGTCTCCAC

L T S L T F F S S L T R V G G L E R L H

1561 ACTGATTACTCCGTGGTGATTCTAGACAACCCCAACTTGCAAGAACTGTGGAACGCGTCA

T D Y S V V I L D N P N L Q E L W N A S

1621 GGCCTCACCATCGAGCGGGGCAAGATTCTGGTCCACGCCAACCCTAAGCTTTGCCTCCAC

G L T I E R G K I L V H A N P K L C L H

1681 CGCGTGCACACACTGGCCAACCAAACGGGCCTCCAGGGCTTGTCCGAGACTGATGTGAGC

R V H T L A N Q T G L Q G L S E T D V S

1741 CCGACCTCTAACGGCGATAAGATTCCTTGTAACATCACGCCGCTGAGAGCCAACGTCAGC

P T S N G D K I P C N I T P L R A N V S

1801 ACCTCGCCCCTGTACGGCACGCTGACGGTGACGGTGGAGGCGACGCAGCTTCCTGGCGTC

T S P L Y G T L T V T V E A T Q L P G V

1861 AGTGTCTACTACGTGAGCTACATGAAGACTGACACCAACATCTCCCTCTACGACAGCGAC

S V Y Y V S Y M K T D T N I S L Y D S D

1921 CAGCCATGTAGCGAGCAGGGATGGGCGATGGTAGAAATGTCAATAGTGAACGGTACTGGA

Q P C S E Q G W A M V E M S I V N G T G

1981 AAAGACGAAGGAAGCATAAGAGAGGAAGGAAGAGGAATAAGAAGTGAAGAAAGAAGAAGA

K D E G S I R E E G R G I R S E E R R R

2041 GGAGGAGGAGGAGGAGGAGGAGGAGGAGGACACATCATCTCCGGCACACTGGTCAACCTG

G G G G G G G G G G H I I S G T L V N L

2101 GATCCATTCACCCGCTACGCCGTTTTTGTCAAGACGGTTTCGCTGGACTCGAGTGCCCGC

D P F T R Y A V F V K T V S L D S S A R

2161 GGTGCCCAGTCCGAGGTGATGTACGCCACCACCAGCGCTTACAATCCCTCGGAGCCTGAG

G A Q S E V M Y A T T S A Y N P S E P E

2221 AACCCGCAGTGGTCATCGCCAAACAGCTCGACGCTCAAGCTGTGGTGGCTGCCCCCCCGA

N P Q W S S P N S S T L K L W W L P P R

2281 CACCCTAATGGCGTGATCGACCACTACCTCGTCACTCTGGTGCTGCTGCTCGACGTCCCC

H P N G V I D H Y L V T L V L L L D V P

2341 AAGATACCCGCTAGCCTTGACTTCTGCATGGAGCAAGAAAGGAGGTACCTTCAGAAAAAC

K I P A S L D F C M E Q E R R Y L Q K N

2401 CTACAGTCCGACCTGGTGATGAGATCCACGTCCTACTCCGACGAAGACGAGATCGAGGAC

L Q S D L V M R S T S Y S D E D E I E D

2461 GAAGAGGACGCGATGGACGAAGAGGAGGAGCACGACGAGGAGGATGACGAGGACGAGGAT

E E D A M D E E E E H D E E D D E D E D

2521 TGGGACAAGGACGTGAGAATGTACAGGAACGAGACGAGAACGGATGGGGACAAAGATGGG

W D K D V R M Y R N E T R T D G D K D G

2581 GAGCTCTCGTGTCGCCAGGCCTCGCACTTCACGGCTTCGTCCTCGTCCTCGTGCTGCGCC

E L S C R Q A S H F T A S S S S S C C A

2641 TGCCCGGAGTCGCCTTCGTCTGAGAGTGACGGGGATGTCCTCGGTCAGATCGCCTTTGAG

C P E S P S S E S D G D V L G Q I A F E

2701 GACTTCATTATGGACAGCGTTTACGTGAAGAAAGCGGGGAGAGGCAGGAGAAGAGCCCGG

D F I M D S V Y V K K A G R G R R R A R

2761 GAGAGACCGCACCAACTGGACGCCTTCGAAAAGAACCTCCGCGCCACCTACCAGCAGCCT

E R P H Q L D A F E K N L R A T Y Q Q P

2821 CGCAGCACCAGACAAGTGCACCACCCAAGGACTTTGGTCTCAAGGAGCTTAGACTCAAGA

R S T R Q V H H P R T L V S R S L D S R

2881 GAGGACCACACGCAGCTTCCTATTGTCTCTGAGGGTGGGGTAATGGAGGAGTATCGTCAT

E D H T Q L P I V S E G G V M E E Y R H

2941 CATCAGCAGCAGTATCACGAGGAAGGGCAAGGAGGTGACTTCCCTTCGTACCCTGAGGAG

H Q Q Q Y H E E G Q G G D F P S Y P E E

3001 TCGGTGCACGAGTTTCCCAGCCAGGGAGGCATAAGGGCAGAGTATCAGCTGCACGAGGGC

S V H E F P S Q G G I R A E Y Q L H E G

3061 GACCTGCACAAGTATAACGAGCCTCTACCCCAGCTTGGTTCAGTCCTCCGCCTTCAGATA

D L H K Y N E P L P Q L G S V L R L Q I

3121 CGCCAGACTCGTAACCTTCACCTCACCATCCACCGCCTGCTGCACTTCTCCCTCTACTCC

R Q T R N L H L T I H R L L H F S L Y S

3181 GTCAGTATCGTGGCGTGTCAGGCACCCGAGCCACTCCTGCCAGACACGTGGATGAAGCTG

V S I V A C Q A P E P L L P D T W M K L

3241 TGTTCCCTCCATCCAGCCCAGACTGCAGCCTACACCAAGCCAAGCGTCGAGGGGGACCAT

C S L H P A Q T A A Y T K P S V E G D H

3301 GTGCGAGCGGGGTCCCTCAAGGTGATGCTACTCAACGGTACCCTGGGCTCCGTCTCTCTC

V R A G S L K V M L L N G T L G S V S L

3361 TCCTGGGAGGCGCCGAGGAGTCCCAACGGAGGGGTGTTGGCTTACGTCATCAGGCTGCAA

S W E A P R S P N G G V L A Y V I R L Q

3421 GGGCCCCTACGGGAGGAGCGGTGCATCACCCACAGGCAGTTCGAGGCGCAGGACGGGCGG

G P L R E E R C I T H R Q F E A Q D G R

3481 CTGGAGCTGGATGACTTGAGCCCCGGGAACTACAGTGTCTCCATCAGGGTCAGGTCCAAG

L E L D D L S P G N Y S V S I R V R S K

3541 GCTAAGTTTGGCCCTTACAGTGAGCCCGTGTTCTTCATCATCCCGGGTCACGCCCCCAAG

A K F G P Y S E P V F F I I P G H A P K

3601 CCAAGCCCTCCTGCCCTTCGCTTGGTACTCTCTGCCGTACTGGGTGTGGTGGGCATGGTC

P S P P A L R L V L S A V L G V V G M V

3661 GCTGCGGGCCTCGGGTGCTGGGGCTGGAGGAGATACCACACTCGTTACACTATCCCTGAC

A A G L G C W G W R R Y H T R Y T I P D

3721 ATCATGGAGGAGGTGGACGAGAACCCCTGCTATCGAGGGGGATTTGCTCCTGCCGAGATG

I M E E V D E N P C Y R G G F A P A E M

3781 TTCCGGCAGGAGTTTATCTTCTGGCGAGACGACCTGAAGGTGCTGCAGGACCGTCCGCTG

F R Q E F I F W R D D L K V L Q D R P L

3841 GGCAAGGGGTTCTTCGGGATGGTGTTTGAGGGAGAACTGACTCACGGGGGCAAGACGCGA

G K G F F G M V F E G E L T H G G K T R

3901 GTGGCGGTGAAGACCCACACGGAGAGGGCCAGCAAGGACGATATACTGCAGTTCCTTAAG

V A V K T H T E R A S K D D I L Q F L K

3961 GAGGCGGCTGTCATGCAGAACATCTCATGCCATCACGTGGTGCGTCTGCTGGGGGTGGTG

E A A V M Q N I S C H H V V R L L G V V

4021 GGCGACTATTCACCGGTCTACGTGGTGATGGAGCTCATGGAACAAGGTGACCTCAAGAGC

G D Y S P V Y V V M E L M E Q G D L K S

4081 TTCCTTCAGCGCAACCCTCGCCACCACGTCTCGGAGGAGAAGCTGGTGGAGATGGCAGTG

F L Q R N P R H H V S E E K L V E M A V

4141 CAGGCGGCTGACGGGATGGCGTACCTAGCAGAGGGGAGGCTGGTGCACCGGGACCTCGCC

Q A A D G M A Y L A E G R L V H R D L A

4201 GCCAGAAACTGTTTGCTGGACCACAACCTTGTGCTTAAGATCGGAGACTTCGGGTTGTCT

A R N C L L D H N L V L K I G D F G L S

4261 CGGAGCCTCAAATCAGACTACTATAGGAAAGAGGGCCAGGGAGTGCTGCCCGTTAAGTGG

R S L K S D Y Y R K E G Q G V L P V K W

4321 ATGGCCCCCGAGAGTCTCCAACGCAGCGTTTATAGCACTCACAGCGACATGTGGAGCTAC

M A P E S L Q R S V Y S T H S D M W S Y

4381 GGAGTGCTGCTGTGGGAGATGGCGACGAGGGGACTCACGCCCTACAAGAACTGCACCAAC

G V L L W E M A T R G L T P Y K N C T N

4441 GAGGAGGTGATCCGGCTGGTGGTGGAGAAGTACGCAACCCTTGACAGGCCGAGGGACAGC

E E V I R L V V E K Y A T L D R P R D S

4501 CCGCAGCCCTTCCAGAGACTCATGCGGCAGTGCTGGCGCTACGACCCTCGAGAGCGCCCC

P Q P F Q R L M R Q C W R Y D P R E R P

4561 ACATTCCTTGCGGTGACCAAGTTTCTCCTGAGGCATACGTCGTCTGAGTTCCAGGAGAGG

T F L A V T K F L L R H T S S E F Q E R

4621 TTCGAAAAAGTGTCCTTCTATCACAGCCGCTCCAGTGACTATGCCAAGTACCGGCAGGCC

F E K V S F Y H S R S S D Y A K Y R Q A

4681 AGCAAGGGCAGCTTCATGAGTGGTGGGGGGCGGGGCAGCGATGATGATGCCTCGGACGAT

S K G S F M S G G G R G S D D D A S D D

4741 ATCGCCCTTGTGTCGTCGTCTGAGAGTGACGAAGAAATCTCGGAAGACGGCGTCCTGCGC

I A L V S S S E S D E E I S E D G V L R

4801 TTCAGGACTCCTCACGGCAGTCCTAGGACCACCAGGAGATCTCCTAGAGGTGCGAAAGGC

F R T P H G S P R T T R R S P R G A K G

4861 GAGATGTCCACCACATTGCTGGGCATGGGTGGCAAGAGTCTGTGCAGGATAGCTGGCGGC

E M S T T L L G M G G K S L C R I A G G

4921 GTGACGGGACCTTCAGACTGGAGCCGCTCCGCCGCGCCTTCCGTCCTGCACTTCTACACC

V T G P S D W S R S A A P S V L H F Y T

4981 AACGTGAGCCCCGACGCGCGGCCTGGCAAGGCAGTGTGTGGGGCGGACCACAGCTCGGCT

N V S P D A R P G K A V C G A D H S S A

5041 GTGGTGCACAGTGCCGGTGAAGACGAGCGGGACCTTCGATACGCTCAGCTGGCACTCAGC

V V H S A G E D E R D L R Y A Q L A L S

5101 ACGCCCAGCACACCAGCCAGTGGGAGTCGCACGCCCCTCCGCACTCCCGGGTCTACGCCA

T P S T P A S G S R T P L R T P G S T P

5161 CACACACCCTTCCTACACCCCAATACAGCCGCGTTCCCCTTGCCGTCTTCCCCAGGTGTA

H T P F L H P N T A A F P L P S S P G V

5221 TCTGGCTGTTCTCCGTCGTTCCTGCAGCCGCATAAAGGAGAAAGCTCTGTCTCGTCGAAG

S G C S P S F L Q P H K G E S S V S S K

5281 AGTCCCTCCCAGGTCAGCCTTGCCACTTCGCCGTCCGCCGCCAAACCCTCAGCGCCATTC

S P S Q V S L A T S P S A A K P S A P F

5341 TACTCAAACTTAGCCAAGTCTTTGTCCACGCTCATCGCTAGGACTTCTGCTCACCCTCAT

Y S N L A K S L S T L I A R T S A H P H

5401 GAGGCGCCACACAGGCTCCTCAGGAGGCTCACCACGGTGCCTCACGGCCTCCGCAGTACC

E A P H R L L R R L T T V P H G L R S T

5461 ACTAACACACTGAAGGACTCCCCCAAGTGGCCTTCTTTCATGGAAGGTCGGAGTAGAACT

T N T L K D S P K W P S F M E G R S R T

5521 CTTCCTTCGTCCACGTCTACATGTAGTGTTCCAACCAACCTCTCTATTGCTGCCCAAACG

L P S S T S T C S V P T N L S I A A Q T

5581 GCGGAACTCACCCACAGCGGATATAGCAGCTCAGAGGTTACGACGCGTGCTCCCTCCATG

A E L T H S G Y S S S E V T T R A P S M

5641 ACGTCCGCAGCTCTTAACGAAGAAACAGTGTCCTCATCCATCCCCAGCATACTCCACGAC

T S A A L N E E T V S S S I P S I L H D

5701 TCGCTGCACTCTTCCTCCAGCAACTCTGAGCACGGAGACGCGGTGCTCACTCTCTCCTCG

S L H S S S S N S E H G D A V L T L S S

5761 AGGCCACAACGATCTAAATCAAAGTTTTTCCGGTGTGAGAGAGTGTACATCAACACGCCA

R P Q R S K S K F F R C E R V Y I N T P

5821 CCAAGAGCCTCAGAAAGCCTCCCAGATAATTTATTCAGAAGACTTCACGCGGAGTACACG

P R A S E S L P D N L F R R L H A E Y T

5881 GAAGCCTTCCTCCCTGCCTCCACAGCAGAGTCTTCCAAGTCGAAAAGCTTGAAGAGATGC

E A F L P A S T A E S S K S K S L K R C

5941 AAAAAGGAAAACGATCCCGGAGACATTGAGATGATG**TGA**AAGGAGAGCAGAAACGCTGAA

K K E N D P G D I E M M *

6001 TACACACACACACACACACACATACACACTCTCACTCTCTCTCTCTCTCTCTCTCTCTCT

6061 CTCTCTCTCTCTCTCTCTCTCTCTCTCTCTCTCTCTCTCTCTCTCTCTCTCTCTCTGTAA

6121 TAACTTTAAGGAACCCTGCGACAGGCAACCTCCTTTTTCTCTCCTCCACACCATCCTCAG

6181 AGGGGCAGGACAGCGGCAGCGTCTCTTAACACCACACATCCGCACCTCGGCCTCATGTTT

6241 CACTCCCAGACACTCAACACTCGCCACAACACTCACAAAGTTCCACACTCAAATTCCTTT

6301 CACTACAACGTTAAAGGAAAACTATCCAATTTCAGAAACATCACGGCAATTCGCTCCTCT

6361 CAGGTGCAAACAATCTCCGCGCCTTGTGACACCTCCCATTCCTTTCCAACGGCGTGGCGA

6421 GGCAATGGAAAATGCATGAACACATTCACCTCCTGTCCTGCTGTCTGTGTGATTCAAGAT

6481 ATGGAGAAGGACGACCAAAGGCCACACAAACACACACGAAGAGAAAAAAAAAAAAAAA

Supplemental S1 The cDNA sequence and predicted amino acid sequence of *Sp-IR*

Start codon (ATG) and stop codon (TGA) are represented in bold font;

Signal peptide: 1-23aa;

—— L domain: 65-180aa, 356-467aa;

------ FU domain: 238-283aa;

FN-III domain: 491-619aa, 634-969aa, 1000-1082aa;

Transmembrane region: 1106-1127aa;

TyrKc: 1172-1427aa.
