# Supplementary material for: Isolation of an Insulin-Like Receptor Involved in the Testicular Development of the Mud Crab Scylla paramamosain
Source: Int J Mol Sci. 2023 Sep 4;24(17):13639. doi: 10.3390/ijms241713639 (PMC10487528; doi:10.3390/ijms241713639)
Supplement: Supplementary file 1 [file ijms-24-13639-s001.zip › ijms-2579348-Table S1.pdf]

Supplementary Table S1. Primers used in this study.

Primer	Primer sequence (5'-3')	Application
IR-F1	CCATCAGGGTCAGGTCCAA	cDNA cloning
IR-R1	CCCGAAGTCGCCGATCTTA	cDNA cloning
IR-F2	CATCAGGGTCAGGTCCAA	cDNA cloning
IR-R2	CGGAGAACAGCCAGATACA	cDNA cloning
IR-3'-1	CTCCCAGGTCAGCCTTGCCACTTCG	3'RACE
IR-3'-2	GGCCACAACGATCTAAATCAAAGTT	3'RACE
IR-3'-3	ACGCCACCAAGAGCCTCAGAAAGCC	3'RACE
IR-5'-1	TCCACCGTTTTCTCTCCTCTACACA	5'RACE
IR-5'-2	GGGAAGTCACCTCCTTGCCCTTCCTCG	5'RACE
IR-5'-3	CTGGCGACACGAGAGCTCCCCATCTTT	5'RACE
IR-OF	ACATCCACCATCGTCCTCACCA	ORF cloning
IR-OR	ACAGACAGCAGGACAGGAGGTG	ORF cloning
IR-qF	AAGCGTCTGTGATGGAAT	qRT-PCR
IR-qF	GGTGTTGAGTGTGGTGTA	qRT-PCR
β-actin-qF	GAGCGAGAAATCGTTCGTGAC	qRT-PCR
β-actin-qR	GGAAGGAAGGCTGGAAGAGAG	qRT-PCR
vasa-qF	TGGCTGCTCGTGGGTTAGA	qRT-PCR
vasa-qR	TCCTGTTCGTCCGATGCG	qRT-PCR
Dsx-qF	CCCACCCATCACTTCACGTT	qRT-PCR
Dsx-qR	GTCACTTCCACCATCCTTCG	qRT-PCR
GFP-F	TGGGCGTGGATAGCGGTTTG	RNAi
GFP-R	GGTCGGGGTAGCGGCTGAAG	RNAi
dsIR-F	AGGACTTCTGCTCACCCTCA	RNAi
dsIR-R	CGCGTGAAGTCGTCTGAATA	RNAi
IR-IF	TCCACCATCGTCCTCACCAC	ISH
IR-IR	CAGCACCACCAGCACCCA	ISH
